# Designing online grocery stores to support healthy eating for weight loss

**DOI:** 10.1017/S1368980021000896

**Published:** 2022-05

**Authors:** Lisa Harnack, Joseph Redden, Simone French, Nancy E Sherwood, Gabrielle Rivera, Sruthi Valluri, Muna Tahir

**Affiliations:** 1 Division of Epidemiology and Community Health, School of Public Health, University of Minnesota, 1300 South 2nd St., Suite 300, Minneapolis, MN 55454, USA; 2 Carlson School of Management, University of Minnesota, Minneapolis, MN, USA

**Keywords:** Internet, Food, Consumer behaviour, Nutrition, Weight loss

## Abstract

**Objectives:**

The current study aimed to identify features to include in online grocery stores to support healthful food purchasing by those striving to lose weight.

**Design:**

A Value Proposition Design approach was used to gain shopper insights, devise potential online grocery store features and obtain feedback on these features.

**Setting:**

Telephone interviews were conducted to gain insight into shoppers’ needs and perceptions. Results were used by the research team to identify potential online grocery shopping features that may support healthful purchase decisions, and interviews were conducted with a different sample of shoppers to gather feedback on features.

**Participants:**

Insight (*n* 25) and feedback (*n* 25) interviews were conducted with convenience samples of adults trying to lose weight.

**Results:**

Participants were primarily female, white, college educated and with obesity or overweight. Online grocery features devised by the research team based on findings from the insight interviews included (1) shopping cart nutrition rating tool; (2) healthy meal planning tool; (3) interactive healthy eating inspiration aisle and (4) healthy shopping preference settings option. Findings from the feedback interviews indicated that the healthy meal planning tool, healthy shopping preference settings option and shopping cart nutrition rating tool features were positively rated by most participants.

**Conclusions:**

There are multiple features grocers should consider including in their online stores to attract and support customers striving to eat healthy for weight loss.

The way that Americans shop for groceries is undergoing a transformation, with leading US food retailers such as Walmart, Krogers and Target now offering online grocery sales for home delivery or pick-up. In 2017, 9 % of Americans reported shopping for groceries online at least once a month and 4 % reported shopping for groceries online at least weekly^(1)^, with steady growth projected^(2)^. More recently, the COVID-19 pandemic led to a sudden large upward shift in online grocery shopping^(3)^, and it is speculated that this change in shopping behaviour may persist beyond the pandemic for some shoppers, leading to greater growth than previously predicted^(4)^. For example, Walmart reported a 74 % increase in e-commerce sales in the first quarter of 2020 in comparison with the first quarter of 2019, and the company’s US CEO believes many of these behaviours will be permanent^(5)^.

As online grocery stores emerge, so too is interest in ways this new shopping platform may be leveraged for public health benefit^(6)^. One line of emerging research is focusing on the potential for online grocery stores to support the needs of those with poor access to brick and mortar supermarkets and grocery stores^(7–10)^. Another area of interest is the potential for online grocery shopping to lead to more nutritious food purchase decisions *v*. shopping in a brick and mortar store due to factors such as less temptation when confronted with a symbolic as opposed to physical representation of a tempting food, fewer sensory cues online (e.g. no food aromas online) and fewer purchases of energy-dense nutrient-poor foods due to making purchase decisions further ahead of potential food consumption^(11–13)^.

There is also interest in identifying ways online grocery stores may be designed to support the unique needs of those striving to eat healthy for prevention and management of chronic diseases. Unlike brick and mortar grocery stores that face physical and logistical constraints with regard to meeting the specific needs of shoppers aiming to make healthy food choices for various reasons, online grocery stores can be more readily tailored to meet individual consumer needs through customisation of the browsing experience. For example, an online grocery store could include nutrition-related search filters that allow shoppers to limit food search results to foods that match the shopper’s preferences (e.g. limit search to foods low in Na). In addition, an online grocer could allow food search results to be sorted by a nutrition attribute of importance to the shopper (e.g. sort ready-to-eat cereals by added sugar content, from low to high).

Currently, research on potential online grocery store features that may support healthy food purchase decisions is limited^(14–18)^. Two studies have evaluated food swaps (offering alternative healthier food options to replace foods in a virtual shopping cart)^(15,17)^. Other studies have evaluated the effect of ranking food search results by a nutrition attribute^(17)^, providing a nutritional quality rating score for foods as well as price reductions for healthier foods^(14)^, and use of available nutrition facts panel and ingredient statement information while shopping for groceries online^(16)^. One study on this topic examined the effect of providing an online shopping basket prefilled with healthy foods on food choices^(18)^.

To build on the limited research conducted to date, we carried out a study that aimed to identify features that could be included in online grocery stores to support healthy food purchase decisions by shoppers striving to eat healthy for weight loss, using a customer-centric approach to product development (Value Proposition Design approach^(19)^) as a framework. Those trying to lose weight were chosen as the target population because many Americans are trying to lose weight. In the 2013–2016 National Health Nutrition Examination Survey, nearly one-half (49·1 %) of US adults reported trying to lose weight within the previous 12 months^(20)^. The Value Proposition Design approach was selected as a framework because it has been contended that approaches that employ design thinking may lead to the development of health programmes, products and services that are more feasible and effective than those developed using traditional approaches^(21–25)^.

## Methods

### Value Proposition Design approach

The Value Proposition Design approach used in the current study is a methodology for developing product ideas (value propositions)^(19)^. The approach is based on the premise that a product development team is more apt to develop a product that meets the needs of customers if a set of tools and processes are utilised that are customer-focused (relentlessly consider customer perspective) and iterative (product design shaped and changed in response to new insights). The approach includes key elements of design thinking, such as being human centred, iterative and relying on prototyping. Although the Value Proposition Design approach was developed for use by companies seeking to increase the odds that a new product succeeds in the marketplace, it has applicability in designing health interventions and technologies. It has been contended that applying design thinking-based approaches in the realm of health interventions and technologies has the potential to lead to the development of health programmes, products and services that are more feasible and effective than those developed using traditional approaches^(21–25)^.

**Fig. 1:**
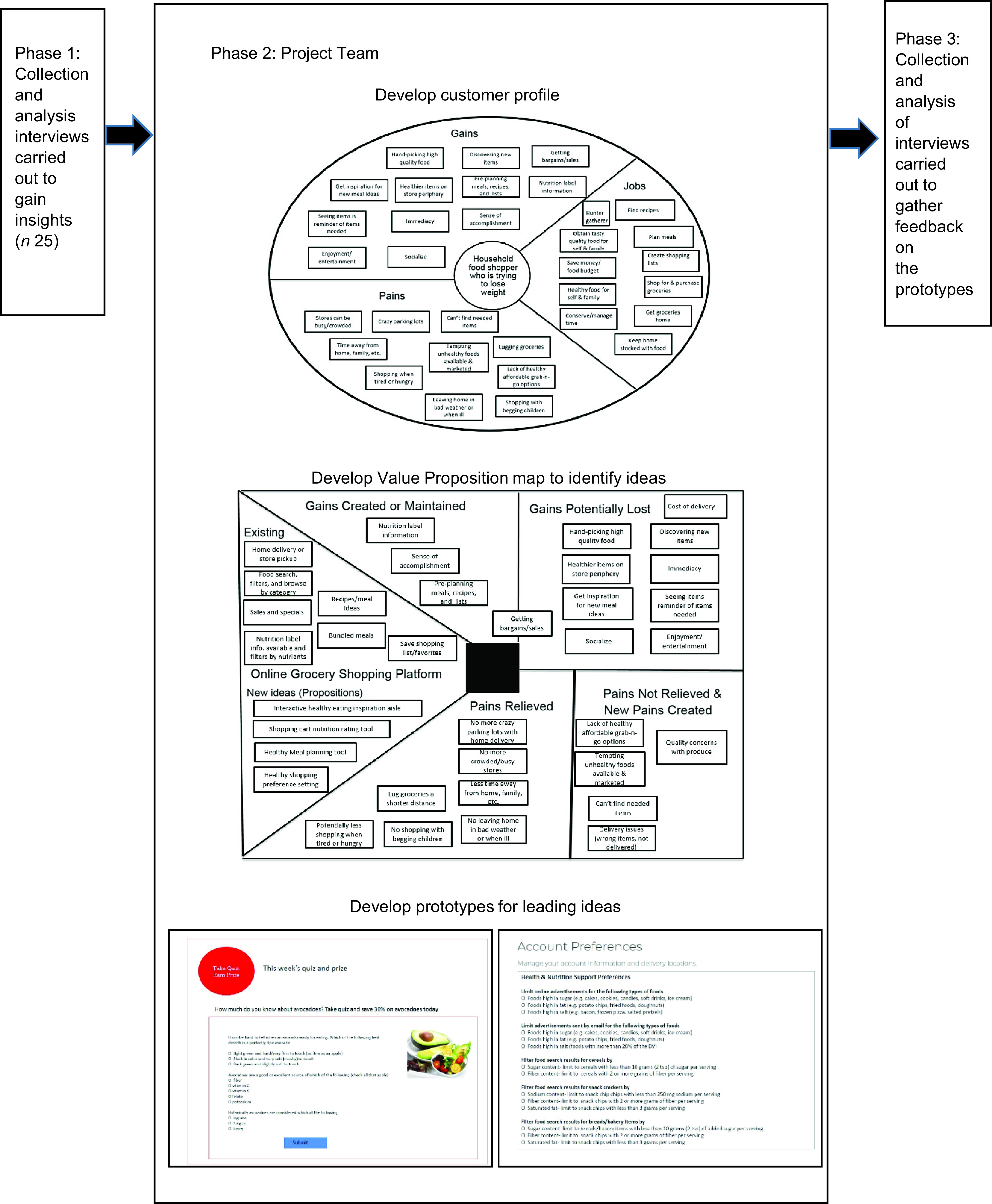
Legend: value proposition design process used in the study

In accord with the Value Proposition Design approach, first a sample of participants were queried about their experiences with retail food store and online shopping (Phase 1-Insight interviews with a community sample). Next, findings from these insight interviews provided the basis for identifying ideas (value propositions) and developing prototypes for the four most promising ideas (Phase 2-Value proposition canvas and prototype development). As a final step, using the prototypes the ideas shared with a sample of participants with feedback solicited (Phase 3-Feedback interviews with a community sample). Each of these activities is described in greater detail below, and a figure is provided to depict the process (Fig. 1).

### Phase 1-Insight Interviews

One-on-one telephone interviews were conducted to collect qualitative data about the experience of shopping in retail food stores, and online settings and participants were asked to complete an online survey. Eligibility criteria were (1) ≥ 18 years of age; (2) primarily responsible for grocery shopping for household; (3) have shopped for groceries online or interested in shopping for groceries online; (4) currently trying to lose weight or maintain weight loss and (5) able to read and speak English. A $35 gift card to a discount retailer was offered as an incentive. Participants were enrolled until convergence was reached (no material new ideas emerging during interviews as determined by one of the authors who listened to the recorded interviews on an ongoing basis).

To recruit study participants ads were placed on Craigslist and Nextdoor, a social media network for neighbourhoods (study ad was posted on several neighbourhood social networks in the Minneapolis-St. Paul, MN metropolitan area). Interested people who contacted the study by phone or email were screened and invited to participate if they met study eligibility criteria. Participants were scheduled for a telephone interview and asked to complete an online survey prior to the telephone interview. Study procedures were approved by the University of Minnesota Institutional Review Board.

The online survey included questions about participant demographics, health history and behaviour related to nutrition and food shopping. Self-reported height and weight were also collected in the survey. The telephone interviews included a series of open-ended questions (see Table 1) aimed at (1) understanding the positive and negative aspects of shopping for groceries for both brick and mortar stores and online; (2) identifying strategies used and barriers to making healthy food purchase decisions when grocery shopping and (3) gathering ideas for ways grocers could improve food shopping and support customers in making healthy food purchase decisions.

**Table 1:** Open-Ended questions asked during the insight interviews

• Tell me about the things you enjoy about shopping for groceries in a store.
• What are some things you dislike about shopping for groceries in a store?
• If you could wave a magic wand to make grocery shopping easier, how would you change grocery shopping?
• How do you define what is a healthy food, and how do you go about evaluating a new food that you are considering buying?
• What strategies do you use to try and make healthy food choices when you are shopping for groceries in a store?
• What things make it hard for you to make healthy food choices when you are shopping for groceries in a store?
• If you could wave a magic wand, how would you make it easier to make healthy food choices when you are grocery shopping?
• Have you ever bought or ordered groceries online, for either delivery to your home or for pick up at the store?
If response is affirmative
○ What online retailers or stores have you ordered through?
○ Describe your experience ordering groceries online.
○ What are some things you enjoy about shopping for groceries online?
○ What are some things you dislike about shopping for groceries online?
○ When shopping for groceries online, how do you go about finding foods on an online shopping site?
○ Describe the ideal online grocery-shopping site that is as easy and automated as possible.
If response not affirmative
○ Have you ever thought about buying groceries online for either delivery to your home or pick up at the store?
If ‘Yes’
○ What has held you back from buying groceries online?
If ‘No’
○ Why haven’t you thought about buying groceries online?
• Thank you for taking the time to talk with me today. Are there any other thoughts you have about grocery shopping that you want to share?

The telephone interviews were audio-recorded and transcribed verbatim, and content analysis was then carried out. Two members of the project team (LH and SV) reviewed the transcripts to identify themes. Responses were then coded into the themes using Microsoft Excel. Coding was carried out by two team members (GR and MT), with discrepancies in coding resolved through consensus decision-making.

Online survey data were analysed using descriptive statistics (means, frequencies, percentages) using Microsoft Excel.

### Phase 2-Value proposition canvas development and prototyping

Using findings from the insight interviews, previous literature and the expertise of the team members, the project team developed ideas (value propositions) for designing an online grocery store to support shoppers in making food purchase decisions that align their health and nutrition goals. To guide idea development the project team developed a Value Proposition Canvas, which is composed of two components: a Customer Profile and a Value Map. The Customer Profile is a structured and detailed description of the experience of the shopper including what they are trying to get done (customer jobs), what shoppers want to achieve/benefits they are seeking (customer gains, and the bad outcomes, risks, and obstacles they face (customer pains). The Customer Profile information then informed the development of a Value Map, which is a structured and detailed description of ideas for designing an online grocery store to support shoppers in making food purchase decisions that better align with their health and nutrition goals. For each idea identified, the team chose pain relievers (how the idea could alleviate customer pains), gains (how the idea could create customer gains) and downsides (how the idea could cause problems for the customer).

One of the team members (LH) was responsible for facilitating discussions related to the Customer Profile and Value Map with the team and drafting the Value Proposition Canvas. To spark ideas and discussion, the team communicated through a series of group email exchanges and an in-person meeting.

From among the ideas the team identified, four leading ideas were chosen. The process of determining the leading ideas involved rank choice voting by team members followed by in-person discussion of the top ranked ideas. Team members were asked to carry out ranking taking into account how useful/successful they thought the idea might be, drawing on findings from the insight interviewers, existing literature and professional judgement in making this determination. Feasibility was not a consideration for ranking or discussion.

After selecting the four most promising ideas, prototypes (mock-ups) were developed for each with the aim of making sure each would be clearly understood when described to the customer. In addition to the prototypes, a brief written description of each of the value propositions was prepared for the ‘customer’.

### Feedback interviews

Feedback Interviews on the VPs were conducted to identify the approaches apt to be most useful and effective in supporting healthy food purchase decisions when shopping for groceries online. To gather feedback on the four Value Propositions, one-on-one qualitative interviews were conducted with a community sample of grocery shoppers who reported that they were currently trying to manage their weight Eligibility criteria for the feedback interviews included (1) ≥ 18 years of age; (2) primarily responsible for grocery shopping for household; (3) have shopped for groceries online in the last two months; (4) currently trying to lose weight or maintain weight loss; (5) able to read and speak English and (6) did not participate in the insight interviews. A $35 gift card to a discount retailer was offered as an incentive. Participants were enrolled until convergence was reached. The same procedures used for the insight interviews were used to recruit participants for the feedback interviews (described above).

As was done with the insight interview portion of the study participants were asked to complete an online survey that was similar in content to that of the insight interview survey. In advance of the one-on-one telephone interviews, participants were sent a copy of the prototypes for the Value Propositions along with a brief written description of each. They were asked to review the prototypes and descriptions before the telephone call and have the prototypes available for reference during the interviews. During the telephone interviews, each idea (value proposition) was described by the interviewer and participants were asked to look at the corresponding prototype while the interviewer described it. Then, the following questions were asked in the order listed: (1) Tell me your general thoughts about this idea. (2) Do you think it would help you make better food choices? (3) Do you have any concerns or things you do not like about this idea that you have not already told me about? (4) Do you have any additional thoughts on this idea that you would like to share?

The interviews were audio-recorded, transcribed and reviewed by two members of the project team (LH and GR) to identify themes that emerged in response to the questions. Responses were then coded by two team members (GR and MT), with discrepancies in coding resolved through consensus decision-making. Responses to the online survey were analyzed using descriptive statistics (means, frequencies, percentages) using Microsoft Excel.

## Results

### Insight interview findings

Twenty-five telephone interviews were completed, with convergence (i.e. no new material themes emerging) reached around the 22nd interview. The mean length of each interview was 18 min 9 s.

Participants were predominantly female (*n* 23), white (*n* 24) and college educated (*n* 21). Most participants had obesity (*n* 11) or overweight (*n* 8) (Table 2). Most participants were trying to make multiple dietary changes (Table 3). The most frequently endorsed goals were ‘lose weight’ (*n* 24), ‘eat less sugar/few sugary foods’ (*n* 24), ‘eat more vegetables’ (*n* 24), ‘limit carbohydrates in the diet’ (*n* 21), ‘limit calories in the diet’ (*n* 18) and ‘eat more fruit’ (*n* 19). When asked to rate the importance of taste, price, nutrition and convenience when buying groceries, most reported taste (*n* 20), price (*n* 15), and nutrition (*n* 14) as very important whereas less than half rated convenience (*n* 9) as very important.

**Table 2:** Demographic and health characteristics of participants in the customer insight (*n* 25) and feedback (*n* 25) interviews

	Customer insight interviews		Feedback interviews	
Characteristics	*n*	%	*n*	%
Sex/Gender				
Female	23	92	20	80
Male	2	8	5	20
Race				
African American	1	4	3	12
White	24	96	20	77
Other	0	0	2	8
Ethnicity				
Hispanic	1	4	2	8
Non-Hispanic	24	96	23	92
Relationship status				
Single	8	32	8	32
Married or partnered	17	68	17	68
Level of education				
High school graduate	0	0	1	4
Some college or associate degree	4	16	4	16
Bachelor's degree	11	44	16	64
Graduate level or professional degree	10	40	4	16
Household size				
1	6	24	8	32
2	7	28	11	44
≥ 3	12	48	6	24
Employment Status				
Work full-time	15	54	13	45
Work part-time	5	18	4	14
Student	2	7	3	10
Retired	1	4	4	14
Working in the home/home maker	3	11	3	10
Unemployed	1	4	1	3
On medical leave	1	4	1	3
Annual Household Income				
< $25 000	2	8	2	8
$25 000–$49 999	3	12	8	32
$50 000–$74 999	5	20	4	16
≥ $75 000	14	56	11	44
Body Weight Status				
Healthy weight*	6	24	7	28
Overweight†	8	32	9	36
Obese‡	11	44	9	36
Health History				
Heart disease or angina	2	8	0	0
High blood pressure	6	24	7	28
Diabetes	3	12	3	12
High cholesterol	9	36	5	20

* BMI (kg/m^2^) 18·5–24·9.

† BMI (kg/m^2^) 25–29·9.

‡ BMI (kg/m^2^) ≥ 30.

**Table 3:** Current dietary goals of participants in the customer insight (*n* 25) and feedback (*n* 25) interviews

	Customer insight feedback		Interviews Interviews	
	*n*	%	*n*	%
Lose weight	24	96	24	96
Eat less sugar/fewer sugary foods	24	96	24	96
Eat more vegetables	21	84	24	96
Limit carbohydrates in diet	21	84	21	84
Limit calories in diet	19	76	20	88
Eat more fruits	19	76	20	80
Eat more protein	17	68	21	84
Limit fat in diet	16	64	16	64
Avoid GM foods	16	64	13	52
Limit salt/Na in diet	14	56	16	64
Eat organic foods	14	56	17	68
Eat more fibre	12	50	22	88
Avoid gluten	4	16	3	12

When asked what they enjoyed about shopping for groceries in a store, one leading theme that emerged was discovering new foods and meal ideas while shopping For example one participant said, *‘I enjoy seeing the new products that are out there, and sometimes things are not necessarily seasonal, but there might be a special purchase or something like that that they don’t typically have, so I enjoy taking advantage of that.’* Picking out quality foods for self, especially produce was another theme. As one participant said, *‘When it comes to produce, I like picking out my own fresh produce v*. *having someone else pick it for me like in terms of an online shopping.’* Bargain hunting/saving money and pleasant shopping environment/experience were other leading themes.

When asked about things they disliked about shopping for groceries in a store, a leading complaint was congestion in the store or parking lot. For example one participant said, *‘If it’s busy and I feel like I have to fight to get a parking spot, and if I have to wait in line for a while.’* Time and effort required, not being able to get what is needed due to being out of stock or not available at the store, and the physicality of shopping (e.g. lugging groceries) were other leading themes.

When asked for ideas for making grocery shopping easier, many ideas raised were price-related. For example one participant said, *‘I would say lower the prices on the fresh produce. Produce is just extremely expensive. So, I think that trying to get your kids to east produce when it costs $6 for a pint a blueberries is ridiculous.’* Other frequently mentioned ideas included reducing congestion (i.e. more parking, streamlining the shopping experience (e.g. more self-checkout), and having tools/ways to make it easier to find items (e.g. better signage or organizing foods differently).

Reported strategies used to make healthy food choices when shopping for groceries in a store included following a set of general food choice rules (e.g. *‘I typically try to stick to like the produce section, the fresh and frozen meats, buying fruits and vegetables, avoiding processed foods.’*). Other leading strategies included planning meals before shopping, reading product labels, making a shopping list, shopping in ‘healthy’ aisles (e.g. produce area), and avoiding ‘unhealthy’ aisles (e.g. *‘I try to avoid like the chip, cracker, candy aisle, so that I’m not even just tempted to put it in the cart.’*).

The most common barriers reported to making health food purchase choices when grocery shopping in stores were merchandizing of unhealthy foods. For example one participant said *‘I think they know exactly what they’re doing and they put a lot of money into the psychology behind where they place products and if I go when I’m hungry, which is right after work, that puts me in a pickle because then I want to buy things that aren’t as healthy.’)*. Visual temptation was also a leading theme. As one participant said, *‘Just seeing food that looks good visually or like at this deli, where they got a rack of ribs or something, they’re hot and ready to go. I mean, I think it’s just tempting or triggers some kind of a thing in me where I’m like, oh, I would love to get some Oreos or I’d love to get nachos. Just seeing food that is probably not healthy makes me want that food.’* Other frequently reported challenges included convenience dilemma (need convenient foods but affordable convenient foods are unhealthy), shopping while hungry, and demands/preferences of family members.

When asked for ideas for making it easier to make healthy food choices when grocery shopping, leading ideas that emerged included increasing the offering of convenient foods that are both healthy and affordable. As one participant said, *“Combine the two: convenience with healthy, because a lot of times convenience means unhealthy, or healthy and convenient is also expensive. I like to have the perfect combination.”* Limiting/removing/hiding unhealthy foods was another leading idea (e.g. *‘I’d probably eliminate food from my vision that is not good for me, so I wouldn’t—that some stuff on the end cap or whatever would not even be there. Just remove unhealthy food from the store”).* Other leading ideas included having healthy foods priced the same as unhealthy and placing ingredients for a healthy meal together in a store (e.g. bundled meal with recipe or meal suggestion with ingredients displayed together).

Those who had previously shopped for groceries online were asked to describe their experience and share things they liked and disliked about shopping for groceries online. Positive aspects of shopping for groceries online included convenience/time savings and the ease with which groceries could be ordered. Lower physical demands (e.g. not having to lug heavy groceries from car to house) was another reported positive aspect of online grocery shopping. The most commonly reported downside of online grocery shopping was lack of control over quality, especially with respect to produce. For example one participant said, *‘The bananas they picked, I didn’t like the size, the color; I don’t know if it was the way there were packaged, they were bounced around. It wasn’t just the bananas; the apples—I didn’t like the color; I was very unhappy with all of it. I’ll never let someone pick my produce for me again.’* Other dislikes included lack of availability of needed items or ordered items missing from delivery, and delivery related issues (e.g. late delivery, improper packaging).

When asked if it was easier or harder to make healthy food choices when shopping online in comparison to shopping in a store, approximately equal numbers thought it was easier, harder or about the same. Reasons it was perceived as being harder were varied, and included reports that it was harder to search for food items online, limited foods available online, and reluctance to buy produce online. Reasons it was perceived to be easier included having more time to make thoughtful decisions (e.g. *‘Because I can take the time to make the decision; I can stick to my plan’*) and less temptation/visual stimuli (e.g. *‘I wasn’t distracted by all the shiny labels of food I shouldn’t be eating’*).

### Value proposition development and prototyping

Using findings from the insight interviews combined with related literature and the expertise of project team members, a Customer Profile was developed. In the Profile the primary jobs a customer is trying accomplish while grocery shopping (e.g. stay within food budget, get healthy foods for family, conserve/manage time) were listed along with pains (e.g. busy parking lot, lugging groceries, tempted by unhealthy foods) and gains (e.g. get inspiration for new meal ideas, hand picking high-quality foods). A Value Proposition Canvas was then developed by the team in which value propositions (ideas) were generated by the team with the aim of devising features that could be included in an online grocery store to support shoppers in making healthy food purchase decisions while alleviating pains and/or creating gains. Potential new pains created and gains lost were also considered in discussing ideas. For example, one of the ideas devised was to develop an interactive healthy eating inspiration aisle that an online shopper could click on to get ideas for healthy affordable tasty meals, learn about new healthy affordable foods, etc. The team discussed how adding an ‘inspiration aisle’ could help shoppers get ideas for healthy affordable meals, a gain. But, visiting the inspiration aisle would likely increase the time it takes to shop for groceries online, thereby creating or amplifying a pain.

Among the ideas identified by the team through the Value Proposition Canvas process, the four most promising ideas were identified through a ranking exercise (each team member ranked the ideas), and in-person discussions held after reviewing results from the ranking exercise. The four leading ideas identified by the team were (1) shopping cart nutrition rating tool, (2) healthy meal planning tool, (3) interactive healthy eating inspiration aisle and (4) healthy shopping preference settings. A brief description of each of the ideas follows.

### Shopping cart nutrition rating tool

This tool provides a nutrition rating of foods in a shopper’s online cart, using a star rating system. As part of the rating tool, suggestions for improving the nutrition quality of one’s cart are provided in an interactive process while shopping (e.g. healthy swaps would be recommended, such as replacing ‘Planters Dry Roasted Peanuts’ with ‘Planters Dry Roasted Lightly Salted Peanuts’). The average rating of other shoppers’ carts is displayed, to provide normative reference with other shoppers (use social comparisons to positively influence purchase decisions). In addition, a shopper’s cart nutrition ratings over time would be plotted so that progress from past food purchases may be tracked by the shopper.

### Healthy meal planning tool

The healthy meal-planning tool supports healthy meal planning and the ordering of foods needed for the meals through a weekly email sent to customers that includes a list of suggested meals tailored to the customer’s personal nutrition goals, food preferences, food budget and family size. For each suggested meal, cost information is provided, along with recipes. The recipe servings are scaled to the shopper’s family size, with links to add ingredients to the shopper’s cart included in the recipe. The shopper must answer some questions about oneself and their family for the meal planning information to be personalised.

### Interactive healthy eating inspiration aisle

The interactive healthy eating inspiration aisle provides an online ‘aisle’ designed to help shoppers discover products and meal ideas that align with their health and nutrition goals. The aisle is designed to be fun and interactive. This aisle includes new product features, free samples and customer reviews; crowd sourced kitchen tips provided through video clips (e.g. video clip on how to cut up a mango); suggested food pairings (e.g. vanilla yogurt + frozen raspberries= instant dessert) and quizzes to build healthy food purchasing and preparation skills (e.g. quiz on how to know when an avocado is ripe enough to eat).

### Healthy shopping preference settings

The healthy shopping preference settings allow an online grocery shopper the option to set - up nutrition-related shopping preferences that prioritise displaying and advertising foods that align with personal health and nutrition goals. A shopper who specifies particular nutrition goals (e.g. limit sugar in the diet, eat more whole grains, limit Na, etc.) will experience an online shopping environment designed to support those preferences. For example, advertisements and discounts that appear (pop-up) while grocery shopping and store advertisements sent by email focus on foods that align with the shopper’s nutrition goals. Foods a shopper is trying to avoid (e.g. foods high in sugar if the shopper is trying to limit sugar intake) are less visible/advertised while online grocery shopping. Filters are available to limit search results by nutrition goals (e.g. allow food search results to be filtered by nutrition attributes such as being ‘whole grain’, ‘low in Na’, etc.).

### Feedback interviews

Twenty five telephone interviews were conducted to gather feedback on the four VP ideas, with convergence reached around the 20th interview. The mean length of each interview was 23 min 26 s. Participants were predominately female, white, and college educated. Most participants had obesity (*n* 9) or overweight (*n* 9) (Table 2). Participants reported several dietary goals (Table 3).

For each idea, participants were asked four questions: (1) share their general thoughts; (2) whether they thought it would help them make better food choices; (3) if they had any concerns and (4) if they had any additional thoughts. Table 4 shows the level of support expressed by participants for each idea when asked to share their general thoughts (open-ended response options were coded as being strongly positive, moderately positive, mixed response, and negative). Three of the ideas (the healthy meal planning tool, the healthy shopping preference settings option and the shopping cart nutrition rating tool) were positively viewed by most participants. Less than half of participants viewed the interactive healthy meal inspiration aisle in a positive way. When asked whether they thought an idea would help them make better food choices, most felt that each idea would help them make better choices (see Table 4).

**Table 4:** Responses to feedback interview questions about general thoughts about each idea (value proposition) (*n* 25)

	Shopping cart nutrition rating tool		Healthy meal planning tool		Interactive healthy eating inspiration aisle		Healthy shopping preference setting option	
Idea/value proposition	*n*	%	*n*	%	*n*	%	*n*	%
General thoughts and concern								
Strongly positive*	11	46	17	68	10	40	11	46
Moderately positive†	6	25	5	20	2	8	8	33
Mixed‡	4	17	3	120	8	32	3	13
Negative§	3	12	0	0	5	20	2	8
Would it help you make better food choices								
Yes||	18	72	20	80	19	79	20	83
No¶	4	16	2	8	5	21	2	8
Unsure/ambivalent**	3	12	3	12	0	0	2	8

* Only positive comments, and used words such as ‘love’, ‘really like’.

† Positive comments using words such as ‘like idea’, ‘OK idea’ with minimal concerns expressed.

‡ Liked parts of the idea but disliked other parts or unsure.

§ Mostly or all negative comments about the idea, using words like ‘dislike’, ‘don’t like’.

|| Thinks it would change some choices.

¶ Does not think it would change any choices.

** Unsure if it will change choices or mixed feelings expressed (e.g. ‘maybe it will, maybe it.

Results for each of the four ideas are presented below.

Shopping Cart Rating Tool: The shopping cart rating tool was positively rated for features such as liking the recommended alternatives/substitutions (e.g. *‘I think the suggestions for what you could swap in and out for different things, I think that definitely would help me.*’), and the ability to track the quality of their purchases over time. Concerns were expressed about the validity of the rating system (e.g. *‘I’m curious about how they would the measure the rating system of healthy foods. That seems relatively subjective based on people’s goals.’*). Also, some disliked the social comparisons. As one participant said, *‘I think I might feel like I’m being compared to other people. The healthy shoppers might be like, “Oh, I’m never going to get to the four-star, like a healthy shopper” and you start might making you feel bad even continuing choosing your choices that—you think you’re doing good, but you’ll never compare.’*

Healthy Meal Planning Tool: For the healthy meal planning tool the predominant themes that emerged included liking the convenience of having suggested meal ideas and recipes, cost/budget (e.g. seeing cost of the meals and recipe ingredients), and tailoring (liked how it may be tailored to personal preferences). *Illustrating multiple themes one participant said, ‘Well, I really love how personalized it is to my nutrition goals and how it goes a step further with taking into account things like my food preferences, my food budget and time constraint, because those are super important to me as well, those factors.’*

Interactive Healthy Meal Inspiration Aisle: The interactive healthy meal inspiration aisle was positively rated with participants liking the offer of free food samples for new food products, liking the suggested healthy food pairings, and feeling it would be interactive and fun. It was less positively viewed as overly time consuming. *For example one participant said, ‘I don’t see myself having a lot of time to do the extra stuff like that. I mean, you’d really have to actually want to do it and take the time to do it. I sit on a computer all day for work. So doing other little tutorials online, sometimes, it is the last thing I want to do at the end of my day because I don’t want to look at a computer anymore.’*

Healthy Food Shopping Preference Setting Online: For the healthy food shopping preference settings option the most common themes that emerged were liking the option to personalize foods/nutrients of interest to them, feeling that it would erase temptation, liking that it would promote healthy foods, and feeling like it would help them keep on track with their diet (e.g. holds you accountable). *A quote from a participant that illustrates a couple of these themes, ‘I think for me personally this is my favorite idea of the four concepts because I don’t want to spend my valuable personal time sifting through things that I’m not going to purchase. I like having the option of setting up—I forgot how you described it, but my preferences for healthy choices so that those are first and foremost. That’s where I’m going to gravitate towards without sifting through the things when I’m really hungry and I’m shopping. Yeah, I think its human nature, you just kind of gravitate towards those comfort foods and high sugar content foods. So if I can eliminate those from my first cart that would be great and just focusing on the good stuff.’*

## Discussion

The current study is the first to use a customer-centric approach to generate ideas for features that may be included in online grocery shopping marketplaces to support healthy food choices for weight loss. Findings suggest there are several features grocers may want to consider incorporating into their online grocery stores to support Americans who are striving to eat healthier for weight loss.

Through the customer insight interviews it was clear that consumers struggle to plan, purchase, and prepare meals that are tasty, nutritious, easy to prepare and affordable. Consequently, it is perhaps not surprising that the meal-planning tool was well liked by most participants in the feedback interviews. Indeed, a large number of meal planning apps are available in the marketplace; however, most do not tie together meal planning, shopping and preparation activities in a cohesive way and are not linked with food ordering^(26)^.

The healthy food shopping preference settings option prototype was also well liked by most, with participants stating they liked it because it would erase temptation (e.g. out of sight out of mind). This finding ties in with a body of literature that indicates that food product placement in grocery stores influences food purchase decisions^(27)^.

The shopping cart nutrition rating tool prototype was well liked by most, but concern with the potential validity of the rating system (how will the ratings be determined?) was raised. This concern connects with real-world challenges in devising a rating system that is both valid and feasible. However, work has been carried out by Brewster et al. to develop and to evaluate a grocery purchase quality index that could potentially be used to rate the nutritional quality of foods in a virtual shopping cart^(28)^.

Some participants in the insight interviews indicated they thought it was easier to make healthier food choices when shopping online due to having more time to make thoughtful decisions and less temptation/visual stimuli. This finding is in alignment with studies that suggest online grocery shopping may lead to healthier food purchase decisions than in brick and mortar stores^(11–13)^. However, it is important to note that some participants thought it was harder to make healthy food choices when shopping for groceries online.

The current study has a number of shortcomings and strengths. Study findings may have limited generalisability due to reliance on convenience sampling and the homogeneity of the resulting study samples (predominately-white, well-educated females who are overweight). Future research should focus on a more diverse population with respect to race/ethnicity, income and gender since needs and solutions likely differ, especially for those with lower socio-economic status. In addition, the study focused on assessing and addressing the needs of adults trying to eat healthy for weight loss. Those striving to eat healthier for other reasons (e.g. blood pressure control, diabetes management and disease prevention) may have a different set of needs and solutions. Study strengths include use of a novel approach to identifying needs and ideas (value propositions) for addressing needs. Design thinking-based approaches may lead to the development of health programmes, products and services that are more feasible and effective than those developed using traditional approaches^(21–25)^. To our knowledge, the current study is the first to report applying this approach to nutrition intervention development.

## Conclusions

With close to one-half of American adults trying to lose weight^(29)^, online grocers have the opportunity to meet the needs of a large market segment by designing their online grocery marketplaces to support healthy food choices. Findings point towards several potential features that may be incorporated into online marketplaces to meet consumer nutrition needs. However, to ensure the needs of a diverse population of consumers are met, further development and evaluation work is warranted. In addition, discussions with food retailers are also needed because there may be marketplace practices, such as product placement agreements with food companies, which place constraints on options.
